# Deep learning approach for denoising low-SNR correlation plenoptic images

**DOI:** 10.1038/s41598-023-46765-x

**Published:** 2023-11-10

**Authors:** Francesco Scattarella, Domenico Diacono, Alfonso Monaco, Nicola Amoroso, Loredana Bellantuono, Gianlorenzo Massaro, Francesco V. Pepe, Sabina Tangaro, Roberto Bellotti, Milena D’Angelo

**Affiliations:** 1 Dipartimento Interateneo di Fisica M. Merlin, Università degli Studi di Bari Aldo Moro, 70125 Bari, Italy; 2 Istituto Nazionale di Fisica Nucleare (INFN), Sezione di Bari, 70125 Bari, Italy; 3 Dipartimento di Farmacia - Scienze del Farmaco, Università degli Studi di Bari Aldo Moro, 70125 Bari, Italy; 4 Dipartimento di Biomedicina Traslazionale e Neuroscienze (DiBraiN), Università degli Studi di Bari Aldo Moro, 70124 Bari, Italy; 5 Dipartimento di Scienze del Suolo, della Pianta e degli Alimenti, Università degli Studi di Bari Aldo Moro, 70125 Bari, Italy

**Keywords:** Optics and photonics, Imaging techniques, Computational science

## Abstract

Correlation Plenoptic Imaging (CPI) is a novel volumetric imaging technique that uses two sensors and the spatio-temporal correlations of light to detect both the spatial distribution and the direction of light. This novel approach to plenoptic imaging enables refocusing and 3D imaging with significant enhancement of both resolution and depth of field. However, CPI is generally slower than conventional approaches due to the need to acquire sufficient statistics for measuring correlations with an acceptable signal-to-noise ratio (SNR). We address this issue by implementing a Deep Learning application to improve image quality with undersampled frame statistics. We employ a set of experimental images reconstructed by a standard CPI architecture, at three different sampling ratios, and use it to feed a CNN model pre-trained through the transfer learning paradigm U-Net architecture with VGG-19 net for the encoding part. We find that our model reaches a Structural Similarity (SSIM) index value close to 1 both for the test sample (SSIM = $0.87 \pm 0.02$) and in 5-fold cross validation (SSIM = $0.92 \pm 0.07$); the results are also shown to outperform classic denoising methods, in particular for images with lower SNR. The proposed work represents the first application of Artificial Intelligence in the field of CPI and demonstrates its high potential: speeding-up the acquisition by a factor 20 over the fastest CPI so far demonstrated, enabling recording potentially 200 volumetric images per second. The presented results open the way to scanning-free real-time volumetric imaging at video rate, which is expected to achieve a substantial influence in various applications scenarios, from monitoring neuronal activity to machine vision and security.

## Introduction

Correlation Plenoptic Imaging (CPI) is a recently established three-dimensional imaging modality that exploits the spatio-temporal correlations of light for enabling plenoptic imaging (PI) at the diffraction limit^1–6^. While in standard plenoptic imaging the required position and direction information are encoded in the intensity registered by a single sensor^7–9^, thus sacrificing image resolution, the volumetric information is retrieved in CPI by measuring spatio-temporal correlations between two disjoint sensors. As a result of the different physical mechanism regulating the two approaches, CPI considerably enlarges the maximum achievable depth of field, at a given resolution, with respect to conventional PI, and significantly improves the volumetric resolution^5,10^. Several alternative configurations of CPI have so far been proposed^4,11–14^, based on the correlation properties of either chaotic light^1^ or entangled photons^3,5^. In all cases, the advantages connected with the use of the spatio-temporal correlation properties of light are counterbalanced by the main open challenge of correlation imaging: the low acquisition speed related with the need for collecting a statistically relevant quantity of samples (i.e., pairs of frames simultaneously acquired by the two sensors) to reconstruct the intensity correlation function. In general, the number of collected frames cannot be reduced too much without negatively affecting the image quality, i.e., its signal-to-noise ratio (SNR)^15^. This trade-off crucially affects temporal performance of CPI, thus limiting its range of effective applicability and its competitiveness with state-of-the-art volumetric imaging techniques, especially when dealing with moving objects. Recently, a first attempt was made by Massaro et al.^16^ to operate CPI at a frame rate approaching video rate: here, correlated photon imaging was demonstrated at a rate of 10 *volumetric* images per second using SwissSPAD2, an array of single photon avalanche photodiodes (SPAD) capable of capturing up to 10^5^ frames per second^14,17–19^. However, to achieve such a frame rate, a trade-off has been made in terms of image quality. The present work addresses this open challenge by developing Artificial Intelligence methods to reduce the number of required frames for extracting effective signals from the typically noisy background of CPI, thus speeding up acquisition while still achieving an established target in terms of image quality.

Artificial Intelligence has led to the widespread use of deep learning (DL) techniques in various fields^20,21^, including image denoising, which has acquired considerable attention. In 2015, Liang et al.^22^ and Xu et al.^23^, used deep networks for image denoising tasks, employing the first Convolutional Neural Network (CNN) architecture for this goal. Later, Mao et al.^24^ utilized multiple convolutions and deconvolutions to suppress noise and restore high-resolution images. Additionally, Zhang et al.^25^ employed the denoising CNN (DnCNN) for image denoising, super-resolution, and JPEG image blocking, through a framework consisted of convolutions, back-normalization, rectified linear unit (ReLU), and residual learning. Considering the tradeoff between denoising performance and speed, Lefkimmiatis^26^ proposed the color non-local network (CNLNet), which combined non-local self-similarity (NLSS) and CNN to efficiently suppress color-image noise. Also in the context of correlated-photon imaging, the use of deep learning techniques has been explored for addressing the problem of noise; a mutual beneficial effect for both imaging speed and image quality has been demonstrated in two scenarios^27–29^: ghost imaging (GI) and computational ghost imaging (CGI). In these contexts, several DL applications have been implemented to increase the quality of the retrieved images with a reduced number of realizations^30–35^, as well as to extend the use of these imaging techniques for tracking moving objects^36^. It is also worth noticing that the exploratory use of Artificial Intelligence techniques is increasingly spreading to other applications of optics and quantum photonics^37,38^.

In this work, we apply DL techniques to address the noise reduction problem in CPI. Despite its similarities with other correlation-based imaging techniques such as GI and CGI, the effect of noise on the measured four-dimensional correlation function is very specific to CPI and its various alternative architectures^15,39,40^. Thus, models developed previously in other contexts cannot be applied directly and a dedicated approach must be developed. We feed our deep model with a sample of refocused images obtained within the experiment performed in Massaro et al.^16^. We employ a model based on the U-Net architecture, where we use, for the encoding part, the convolution section of a pretrained VGG-19 net, thus realizing a transfer learning model to improve the denoising power. To demonstrate the effectiveness of our deep model, we compare our results with those obtained by using a combination of two well-known image noise-reduction filters, namely, bilateral^41^ and Gaussian filters. This work represents the first application of an artificial intelligence method to the field of correlated photon-based plenoptic imaging, and paves the way for extending CPI to scan-free real-time volumetric imaging at video rate.

**Figure 1:**
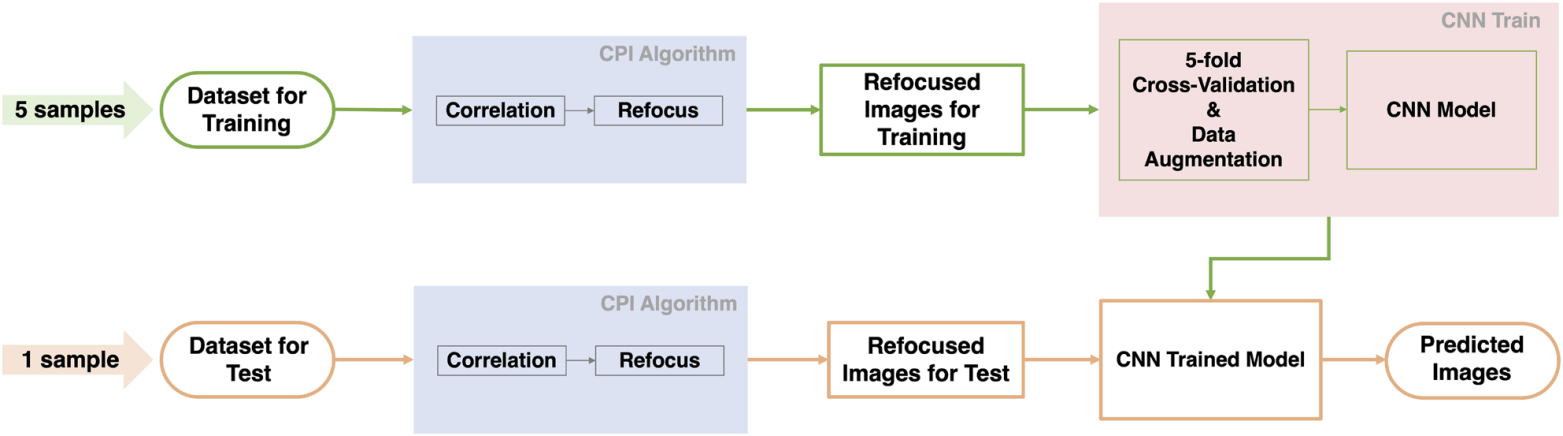
The flowchart of CPI using convolutional neural networks (CNNs). Green represents the training stage and orange the testing stage. The steps composing the analysis and the related findings are described in detail in the “Results” and “Materials and methods” sections.

## Results

We adopted a DL strategy to mitigate noise in CPI. To increase the de-noising capacity of our system, we used a transfer learning approach with a model in which we combined a U-Net architecture and a popular deep CNN named VGG-19 (see “Materials and methods” for details) that were previously trained through the ImageNet database. A scheme of the workflow implemented in the present research is displayed in Fig. 1. We exploited captures of 6 different planar transmissive targets to obtain as many 128x128 pixels refocused images. Then, for each target, we produced three sets of undersampled refocused images (100 images for each set) using three different sampling ratios (*S*) (0.025%, 0.25% and 5%). So, after the image generation procedure, we obtained 6 data sets containing one hundred 128x128 pixels images for each *S*. At a fixed *S*, we used 5 generated data sets to train the network within a 5-fold cross validation (CV) procedure and the sixth data set to test the model. Inside the 5-fold CV framework, repeated 100 times, we applied a data augmentation procedure on 4 of 5 data sets for a total of 4400 training images after the data augmentation procedure and 100 images used to validate the model. Figure S2 of the Supplementary Materials section shows loss and learning rate as a function of epochs for a single network implementation. To estimate the quality of the output images, we evaluated their Structural Similarity (SSIM) with their respective labels. Further details are given in the “Material and methods” section. The result of 5-fold procedure are reported in Table 1 where SSIM ranging from 0.63 for $S = 0.025\%$ to 0.92 for $S = 5\%$. We reported computational network parameters in Table S1 of the Supplementary Materials section.

**Table 1:** Summary of the performance measure (SSIM) of the proposed deep learning (DL) approach, as obtained through a 5-fold cross validation procedure repeated 100 times.

Sampling ratio (S)	SSIM label-input	SSIM label-output
0.025%	0.050 ± 0.001	0.630 ± 0.150
0.25%	0.090 ± 0.010	0.680 ± 0.140
5%	0.280 ± 0.010	0.920 ± 0.070

The SSIM values obtained for the 3 considered sampling ratios are reported with the corresponding standard deviations. Input refers to images obtained by CPI; output to images obtained after implementing our DL model.

**Figure 2:**
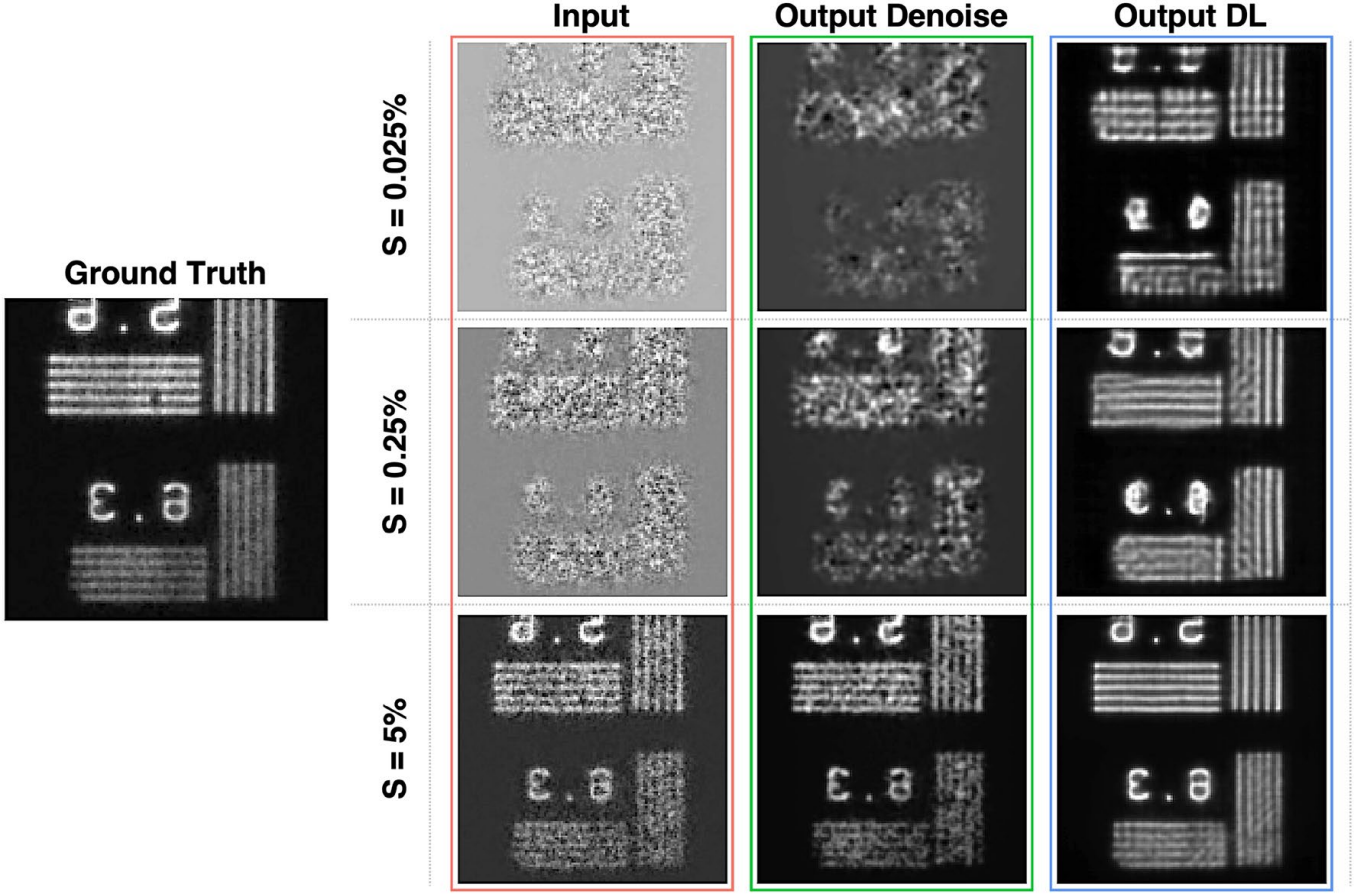
The panel shows a qualitative comparison of the output of our algorithms applied to the test dataset. Starting from the left we see: the Ground Truth image, the input images obtained by the standard undersampled CPI algorithm, the output of the denoising method and the output of DL. *S* stands for the sampling ratio.

**Figure 3:**
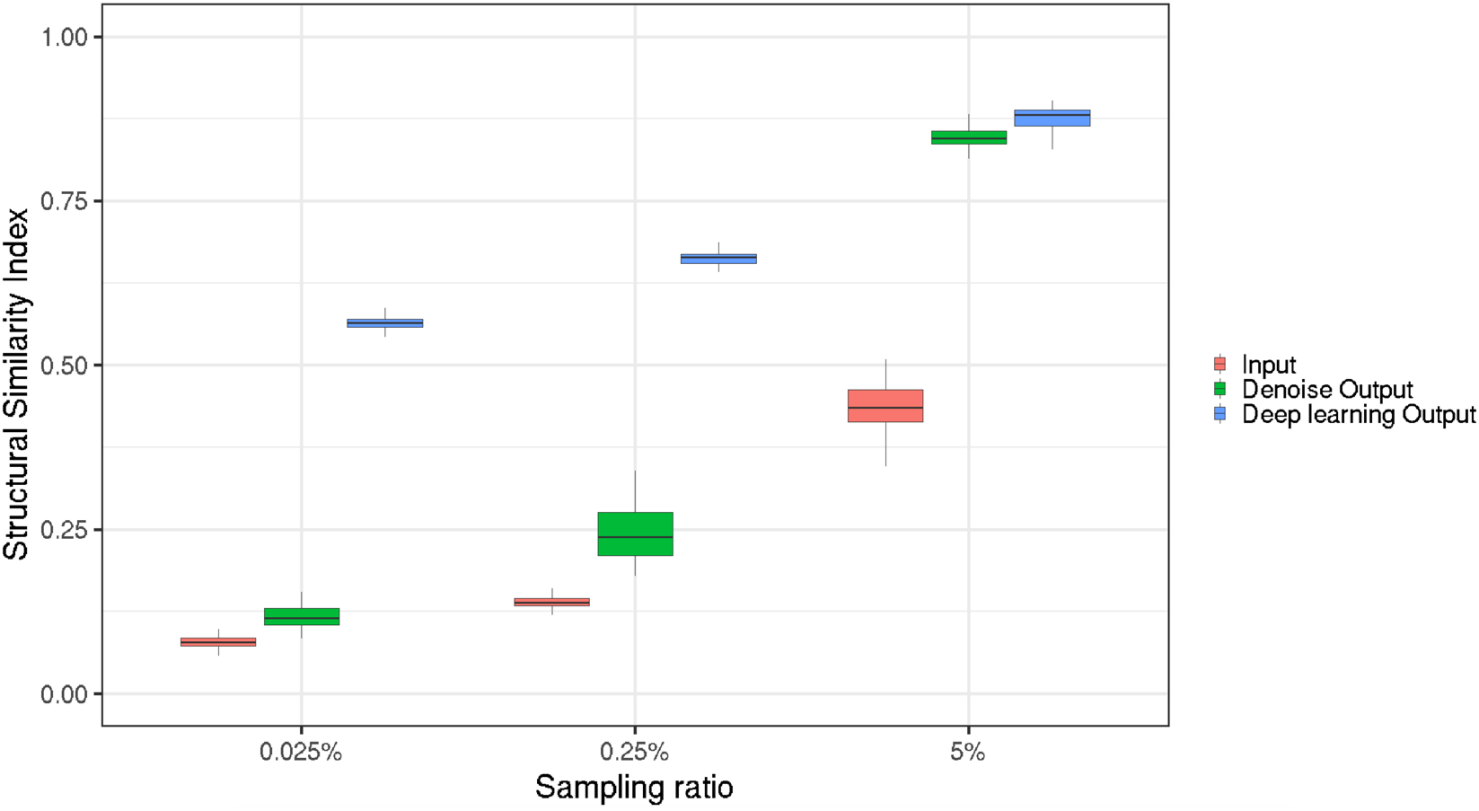
SSIM distributions for the test sample and for the three different sampling ratios so far considered. We computed SSIM (with respect to the ground truth image) for the input CPI images (red), the images obtained with the standard denoise procedure (green) and those from DL (blue).

Then we tested the trained model through the sixth data set of generated images. Figure 2 shows a qualitative comparison between an image of the test sample obtained by the standard CPI refocusing algorithm in different *S* conditions (Input), the corresponding output images of the conventional denoising and the outputs of DL models. By visual inspection, starting from $$S=5\%$$, we can see that the image reconstructed by the deep neural network, is almost identical to the ground truth image. At a lower sampling ratio ($$S=0.25\%$$), DL algorithm provides a reasonable reconstruction of the target. For an even lower sampling ratio of $$0.025\%$$, we get only a partial reconstruction of the target, but the result is excellent when compared with the initial CPI image. Our denoising strategy (Bilater + Gaussian filters) is able to reconstruct the image with a good yield only in the case of the highest sampling ratio. These findings are confirmed by applying another well-known denoise algorithm, named Block-matching and 3D (BM3D) filter^42^, as shown in Fig. S6 in the Supplementary Materials section. Furthermore, to state the rebustness of our results, we show more reconstructions of the test dataset in Figs. S3, S4 and S5. The capability of CNNs to reconstruct much clearer images can also be observed in Fig. 3, where we report, for each sampling ratio, the distributions of SSIM (computed with respect to the ground truth) obtained by applying both our DL framework and the standard denoising strategy to the test sample. DL resulted the best performing method, providing SSIM ranging from 0.46 for $$S = 0.025\%$$ to 0.87 for $$S = 5\%$$, and, according to a Kruskal–Wallis test^43^, the two methodologies of noise reduction are significantly different ($$p < 0.1)$$ for each considered *S*.

## Discussion

As mentioned earlier, our DL model was trained using experimental images taken at a specific setting, based on the CPI architecture and employing SPAD arrays as sensors as implemented in Massaro et al.^16^. In these refocused images, noise inherent to the image formation procedure, i.e., the computation of pixel-by-pixel intensity correlations, prevents a standard denoising algorithm from significantly increasing the SNR of the image. Our DL model significantly improves the performance with respect to the standard denoising algorithm despite the small training sample. A well known limitation of DL algorithms is that they have poor performances when the number of observations in the training set is too low and thus not informative enough. Therefore, to improve the performance of our model, it would be necessary to have a larger number of images in the training phase, for example by using simulated images based on realistic noise models. Through simulations, the algorithm can also be trained on a richer variety of possible scenarios and objects that are difficult to deal with experimentally, such as complex objects requiring a much larger statistical pool than a ground glass disk, or fast-moving objects. Furthermore, the use of more complex simulated images is reasonably expected to increase the discriminative power of the deep model.

It is worth remarking that a sampling ratio of 5% allows to perform CPI at 10 Hz with a satisfactory SNR level, as demonstrated by the SSIM reported in Fig. 3. However, the application of our DL model enables to obtain SNR values fully comparable with the ground truth, as demonstrated in Fig. 2. This primarily leads a general improvement of image quality under condition of low-SNR, but more specifically it shows that it is possible to further reduce the required number of frames and achieve video rate acquisition speed, as demonstrated by the cases with a lower sampling ratio in Fig. 2. In fact, if we take into account the actual frame rate of the SPAD array used in our experiment (almost 100.000 frames per second), it is possible to estimate a potential acquisition speed of the CPI setup of 200 volumetric images per second for $S=0.25\%$, and even 2000 volumetric images per second for $S=0.025\%$, therefore more than a factor of 20 compared to the fastest CPI demonstrated so far.

A direct comparison of our results with the literature is difficult to make, because our work is the first application of a DL model to CPI. However, as mentioned in the “Introduction”, DL methods for noise reduction have been applied to ghost imaging. In particular, for computational ghost imaging (CGI), Rizvi et al.^33^ used deep convolutional autoencoder network to achieve imaging at a frame rate of 4–5 Hz with 10–20% sampling ratios, for reconstructing good-quality $96\times 96$ images. Earlier, Lyu et al.^30^ and He et al.^31^ used DL approaches, also with the support of Compressive Sensing^44^, to reconstruct, still in the CGI framework, good-quality 32x32 and 64x64 images, respectively, with sampling ratios between 5 and 20%. Recently, Hu et al.^36^ demonstrated the possibility to reconstruct both the trajectory and a clear image of a moving object via GI, by using a convolutional denoising auto-encoder network; the quality of images was enhanced with a sampling ratio even down to 3.7%. Our work shows that the employed DL method can achieve a sampling ratio smaller than 1%, with an SSIM of about 0.7. As mentioned before, this result demonstrates the potential of our approach to retrieve volumetric images at a frame rate larger than 200 Hz, well beyond video rate.

We acknowledge that our work presents some other limitations. Mainly, in the training phase of our DL model, the computational demands, in terms of RAM, GPU and computation time, rapidly increase with image resolution. So using higher image resolution (for example $1024\times 1024$ pixels) would require a different approach (e.g. image patch calculations).

Further research will be dedicated to the application of Artificial Intelligence methods directly on the images acquired by the two sensors of CPI setup, in the attempt to entrust DL with both the data analysis, and the denoising stage, by feeding the algorithm the raw data and obtaining the denoised 3D stack of refocused images as the output. This type of approach could revolutionize the CPI technique, because it would definitely overcome the problem of image acquisition and reconstruction times that currently represents the main bottleneck towards real-time volumetric imaging.

## Materials and methods

### Correlation plenoptic imaging

The dataset used to train and test the network is composed of images acquired in a setup based on the concept of *correlation plenoptic imaging between arbitrary planes*^13^ (CPI-AP). Massaro et al.^16^ describes both its working principle and experimental realization in detail: the conjugate planes of the two high-resolution sensors are located at general axial distances from an imaging lens, in the surroundings of the object of interest. A beam-splitter is used to deflect the chaotic light from the object onto the two sensors. Unlike a conventional light-field camera, which uses both the usual camera lens and a microlens array, our setup is implemented with a single lens that captures light from the selected planes and focuses it on the sensors. To avoid the need for synchronization, the two sensors are realized by using two halves of the same SwissSPAD2 sensor^17,18^; each acquired frame, thus, consists of a binary matrix identifying the pixels triggered by at least one detected photon. The software evaluates the correlations between the photon-number fluctuations, pixel by pixel, between the two halves of the sensor, and reconstructs the volumetric image of the scene. As the light from the scene is chaotic, by calculating the simultaneous pixel-by-pixel correlation between the number of photons detected by the sensors, we obtain the correlation function:

1 $$\begin{aligned} \Gamma (\varvec{\rho }_a,\varvec{\rho }_b) = \langle N_a (\varvec{\rho }_a) N_b (\varvec{\rho }_b) \rangle - \langle N_a (\varvec{\rho }_a) \rangle \langle N_b (\varvec{\rho }_b) \rangle , \end{aligned}$$

where $N_a$ ($N_b$) and $\varvec{\rho }_a$ ($\varvec{\rho }_b$) are the number of photons and the coordinates denoting the pixel positions on the sensors *a* (*b*) respectively, while $\langle \dots \rangle$ indicates the averaging process. The correlation function in Eq. (1) represents the correlation between the intensity fluctuations reaching two points, one placed on the first, and the other on the second detector. $\Gamma (\varvec{\rho }_a,\varvec{\rho }_b)$ contains plenoptic information and thus allows the reconstruction of features of a 3D object that can lie both between and beyond the two selected planes imaged on the detectors^12,45^. $\Gamma (\varvec{\rho }_a,\varvec{\rho }_b)$ encodes a collection of multi-perspective volumetric images; proper processing of these volumetric images provides the *refocused image* of a specific transverse plane in the scene.

#### Image generation

**Figure 4:**
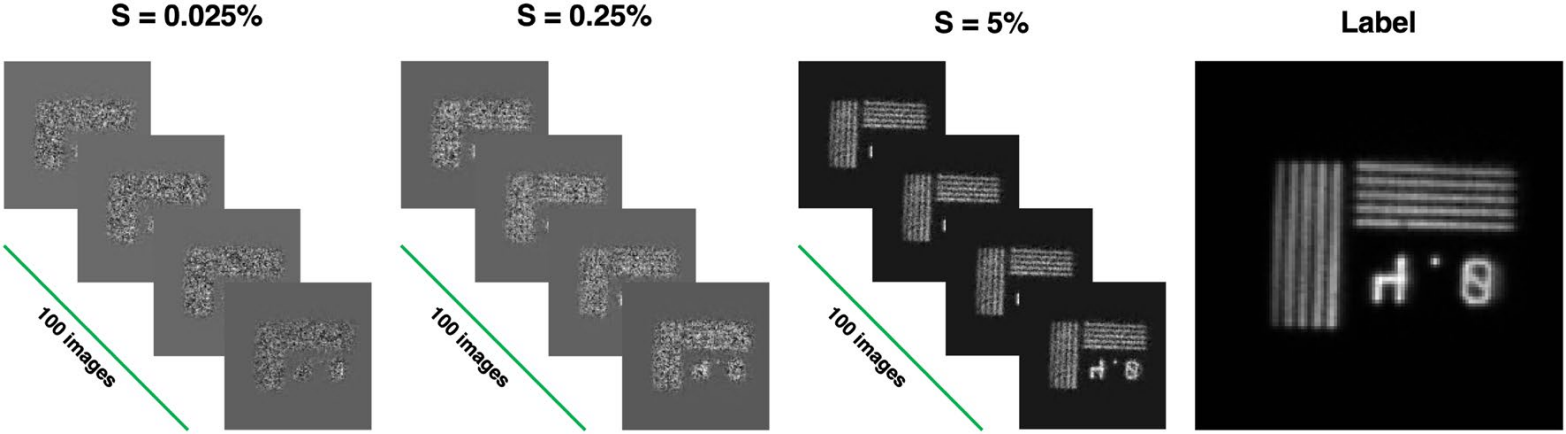
Schematic representation of the construction of each data set: for each sampling ratio *S*, 100 images were reconstructed, each one containing a different random noise due to the random choice of the frames from the acquired out-of-focus images of the test target.

In our specific case, the CPI device is used for imaging several transmissive planar test targets placed out of focus on both sensors. The targets are illuminated by a chaotic light source with controllable polarization, intensity, and coherence time, generated by a laser scattered by a rotating ground glass disk. We performed a series of acquisitions with the object at different axial positions. For each target, we acquired a large number of frames (larger than 200 k). Following the workflow of the refocusing algorithm^45,46^, we used these data to create a dataset for training and testing the network. Basically, we used 6 acquisitions of different transmissive targets and we exploited each full dataset to achieve a $128\times 128$ pixels refocused image of the sample. The retrieved images can be considered as our *ground truth*. However, the behaviour of the SNR of a refocused image deviates from the $\sqrt{N_t}$ scaling, where $N_t$ is the number of the acquired frames, since our source can only provide a finite number of statistically independent realizations^16^. For this reason, we estimated the full sampling rate at 200 k detections. Thus, we define the sampling ratio *S* as the ratio between the considered *number of acquired frames* and *the total number of acquired frames at the full sampling rate*. To test our DL approach, we generated three sets of undersampled refocused images, for each considered test target, using three different sampling ratios *S* (0.025%, 0.25% and 5%). For a given value of the sampling ratio *S*, each data set was generated by randomly extracting, from the images of the target directly retrieved by the sensor, a number of frames corresponding to the considered sampling ratio. By repeating the random procedure 100 times, we were able to increase the variability of the data sets while keeping constant the introduced noise level. Figure 4 schematically shows the composition of each dataset: 5 datasets of $128\times 128$ pixels images have been used to train the network for each value of *S*, and the remaining one has been used for testing the model. It is worth noting here that all the data sets were built in the same modality, namely, starting from images of different targets placed in different axial positions within the same setup described in the dedicated section.

### Deep learning

After the image collection phase we developed a DL model based on CNN framework to remove image noise. Figure 1 shows a schematic overview of the performed analysis: first, we trained 3 DL models, one for each sampling ratio used (see “Image generation” section for details), then we tested our algorithms on a dataset independent of the training sample.

#### Data augmentation

When there is only a limited number of training samples available, it is crucial to use data augmentation techniques in order to train the network on the required invariance and robustness characteristics. Therefore, we implemented Data Augmentation by rotating the source images at various angles, and also using the transposed image and its rotations. Specifically, for each training image we built: 6 rotations (45°, 90°, 135°, 180°, 225° and 270°), the transposed image, 3 rotations of the transposed image (90°, 180°, 270°). In this way, our training set has reached the number of 4400 images (from the initial 400) for each sampling ratio used.

#### Learning model

CNNs are a class of DL algorithms that have been specifically developed to address various computer vision and image processing tasks^47,48^. CNNs structure is inspired by the visual cortex of some animals^49,50^, and is comprised of three main types of layers: convolutional, pooling, and fully connected layers. In contrast to conventional Artificial Neural Networks, CNNs eliminate the need for a feature engineering and extraction process as the convolutional layer automatically performs these functions. This layer uses both linear and nonlinear operations by applying a fixed-dimension filter (known as a kernel) across the layer’s input during each linear operation. The resulting output is then transferred to a nonlinear activation function. The pooling layer conducts a downsampling operation over the feature maps’ spatial dimensions. The most frequently utilized pooling operation is max pooling, which extracts fixed-dimension blocks from the input feature maps and retains only the maximum value in each block. Over the last years, deep convolutional networks overcame previous best practices in various visual recognition assignments. In particular, the newly suggested CNNs have significantly improved the removal of image noise procedures because of their powerful expressive capabilities and speedy performance^51^. In this work, we used a transfer learning approach with a model composed by two different algorithms (U-Net and VGG-19 architectures) for a noise reduction task. The U-Net is a type of CNN that was created specifically for the purpose of biomedical image segmentation in 2015^52^. Unlike typical CNNs, the U-Net is designed to be trained using a smaller number of images. Its architecture consists of four coding blocks connected to four decoding blocks via a bridge and four “skip connections” that bring directly in the decoding block the spatial information from the encoding blocks. The encoding part works as a feature extractor and learns, during the training phase, an abstract representation of the image, which the decoding part expands back to its initial size. By using noisy input images and corresponding clean images as labels during the training phase, U-Net can learn to denoise similar images within the same domain as the input images. The VGG-19^53^ is a DL model consisting of 19 layers, of which 16 layers are convolutional and the remaining 3 are fully connected. Its main goal is to classify images into 1000 different categories using the ImageNet database, which includes a vast collection of images. The 16 convolutional layers are employed for feature extraction and are divided into five groups, each followed by a max-pooling layer. Finally, the last three layers of the model are used for classification. In this work we used the U-Net architecture, with the encoding part formed by the pre-trained VGG-19 Feature Extraction block. In our configuration we implemented the default parameters: the binary crossentropy loss and Adam optimizer. The U-NET was composed by a final convolutional layer with 1 filter, 1 pixel kernel and sigmoid as activation function. Starting from the weights of VGG-19, pretrained on ImageNet database, we trained our DL model through a cross validation procedure, as detailed in the following section. We reported the network structure in Fig. S1 of the Supplementary Materials section.

#### Cross validation

To increase the robustness of our DL model, we implemented a 5-fold cross validation (CV) framework using 5 of the 6 generated data sets. It is worth emphasizing that the further data set (the sixth) has been used to provide an external validation of the DL algorithm. This technique entails partitioning the original data set into five non-overlapping subsets consisting of the same number of cases, which are assigned to each fold on a random basis. We employed four of the five subsets for training purposes and reserved the remaining portion for validation. On the training sample we applied the data augmentation procedure described in a previous section. We repeated CV 100 times so the average of the 100 performance values is a reliable indicator of the overall model accuracy.

#### Performance metrics

As we anticipated, refocusing images starting from an undersampled correlation function leads to a worsening of the image quality due to statistical noise; in fact, it is well known that the SNR of correlation-based imaging techniques improves with the square root of the number of correlated frames. In CPI, however, attributing a single numerical value to the statistical SNR for estimating the image quality can be ambiguous: from Eq. (1), we see that the SNR of the correlation function, defined as the correlation function itself over its statistical variance, is a local non-homogeneous four-dimensional quantity, depending on all four coordinates at the detectors. Because of its local nature, the SNR cannot be used as a global image quality estimator for the refocused images as is. Here, we choose a straightforward approach, that is to assess the quality of our image reconstruction in terms of Structural Similarity (SSIM) index^54^, used as a proxy of the statistical SNR, which has the advantage of being a global estimator for the image quality. This index quantifies the degradation of structural information in an image and evaluates the similarity measurement through 3 comparisons: luminance, contrast and structure. Given an image $\varvec{x}$ considered to have perfect quality, we can measure quantitatively the quality of a second image $\varvec{y}$ by means of a similarity measure with $\varvec{x}$,

2 $$\begin{aligned} SSIM(\varvec{x}, \varvec{y}) = [l(\varvec{x}, \varvec{y})]^{\alpha } \cdot [c(\varvec{x}, \varvec{y})]^{\beta } \cdot [s(\varvec{x}, \varvec{y})]^{\gamma } , \end{aligned}$$

where $\alpha$, $\beta$ and $\gamma$ are positive parameters used to modify the relative importance of the three components. The first term in Eq. (2) indicates the luminance comparison

3 $$\begin{aligned} l(\varvec{x}, \varvec{y}) =\frac{2\mu _x\mu _y + C_1}{\mu _x^2 \mu _y^2 + C_1} , \end{aligned}$$

with $\mu _x$ and $\mu _y$ the mean intensity of $\varvec{x}$ and $\varvec{y}$ respectively and $C_1$ a constant. The second term in Eq. (2) represents the contrast comparison function

4 $$\begin{aligned} c(\varvec{x}, \varvec{y}) =\frac{2\sigma _x\sigma _y + C_2}{\sigma _x^2 \sigma _y^2 + C_2} , \end{aligned}$$

that is expressed as the comparison between the standard deviation of $\varvec{x}$ ($\sigma _x$) and $\varvec{x}$ ($\sigma _y$). The last component of Eq. (2) defines the structure comparison function

5 $$\begin{aligned} s(\varvec{x}, \varvec{y}) =\frac{2\sigma _{xy} + C_3}{\sigma _x \sigma _y + C_3} , \end{aligned}$$

where $C_3$ is a constant and $\sigma _{xy}$ is

6 $$\begin{aligned} \sigma _{xy}=\frac{1}{N-1}\sum _{i=1}^N (x_i - \mu _x)(y_i-\mu _y). \end{aligned}$$

### Conventional denoising

To demonstrate the effectiveness of our approach, we compared the results with the ones achieved by combining two conventional denoising approaches: Bilateral filter^41^, and a Gaussian filter. The bilateral filter is a well known type of non-linear noise reduction filter having the peculiarity to preserve edges; it has been used in the most diverse contexts, including correlation imaging^32^. Here, it was applied to the reconstructed testing images. Because of the binary nature of the target used in the experiment (i.e. a negative transmissive resolution mask), a Gaussian filter was then applied to the previously filtered images in a minimally invasive way, with the aim to close the possible artificial gap between adjacent pixels^55^ inside the correlated regions. We applied this combined noise reduction method to each image of the same data sets generated under the three sampling ratio conditions used for testing our DL model.

### Supplementary Information

Supplementary Information.

## Data Availability

The datasets used and/or analysed during the current study are available from the corresponding author on reasonable request.
