# Supplementary material for: Deep learning approach for denoising low-SNR correlation plenoptic images
Source: Sci Rep. 2023 Nov 10;13:19645. doi: 10.1038/s41598-023-46765-x (PMC10638444; doi:10.1038/s41598-023-46765-x)
Supplement: Supplementary file 1 — Supplementary Information. [file 41598_2023_46765_MOESM1_ESM.pdf]

SUPPLEMENTARY MATERIALS
Deep Learning approach for denoising low-SNR correlation plenoptic images

Francesco Scattarella[1,2], Domenico Diacono[2], Alfonso Monaco[1,2,*], Nicola Amoroso[2,3], Loredana Bellantuono[2,4], Gianlorenzo Massaro[1,2], Francesco V. Pepe[1,2], Sabina Tangaro[2,5], Roberto Bellotti[1,2,†], Milena D'Angelo[1,2,†]

[1] Dipartimento Interateneo di Fisica M. Merlin, Università degli Studi di Bari Aldo Moro, 70125 Bari, Italy
[2] Istituto Nazionale di Fisica Nucleare (INFN), Sezione di Bari, 70125 Bari, Italy
[3] Dipartimento di Farmacia - Scienze del Farmaco, Università degli Studi di Bari Aldo Moro, 70125 Bari, Italy
[4] Dipartimento di Biomedicina Traslazionale e Neuroscienze (DiBraiN), Università degli Studi di Bari Aldo Moro, 70124 Bari, Italy
[5] Dipartimento di Scienze del Suolo, della Pianta e degli Alimenti, Università degli Studi di Bari Aldo Moro, 70125 Bari, Italy
[†] Co-last authors
[*] Corresponding author: alfonso.monaco@ba.infn.it

Table S1: Network Parameters

**Parameters**	**Value**
Number of trainable parameters	31168193
Starting learning rate	0.001
Batch size	20 images
Training time (5-fold CV repeated 100 times)	13 hours
Training time (single model)	5 minutes
Computational resources	NVIDIA A100 GPU with 40GB RAM

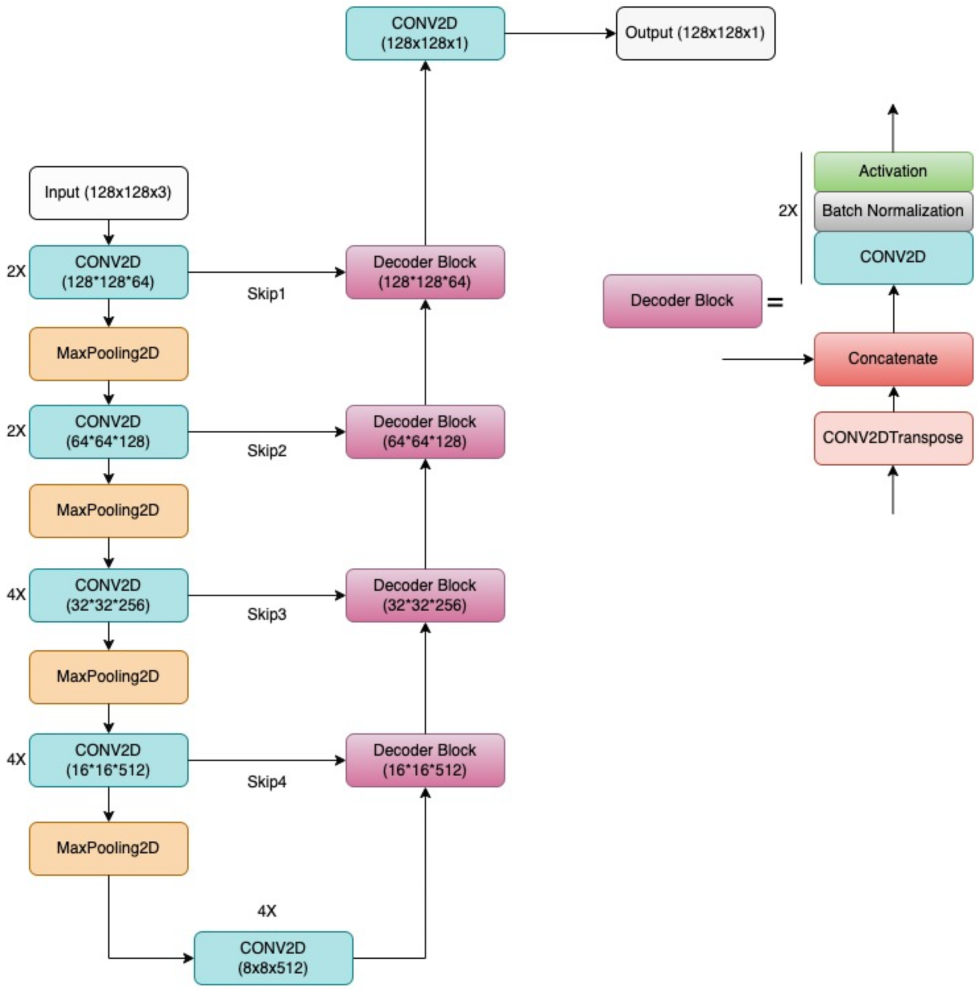

Figure S1: Network structure diagram

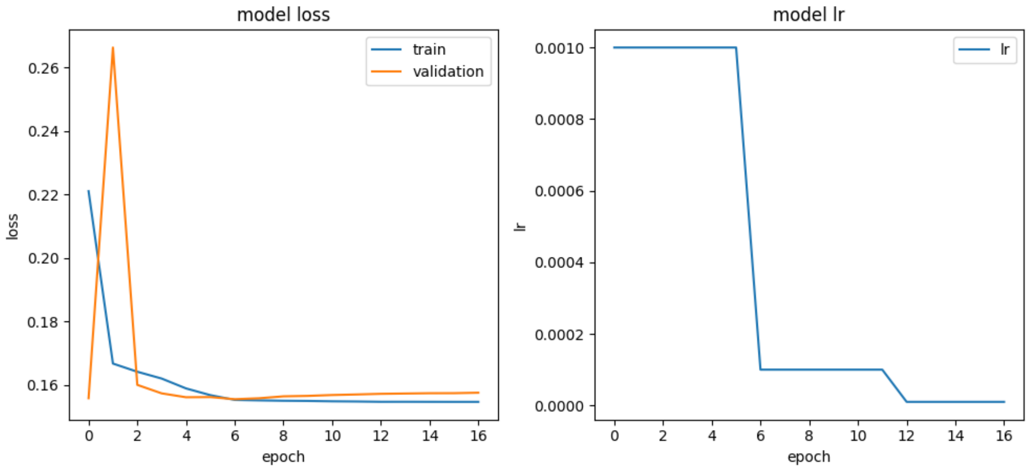

Figure S2: Loss function (left panel) and learning rate (right panel) as function of epochs.

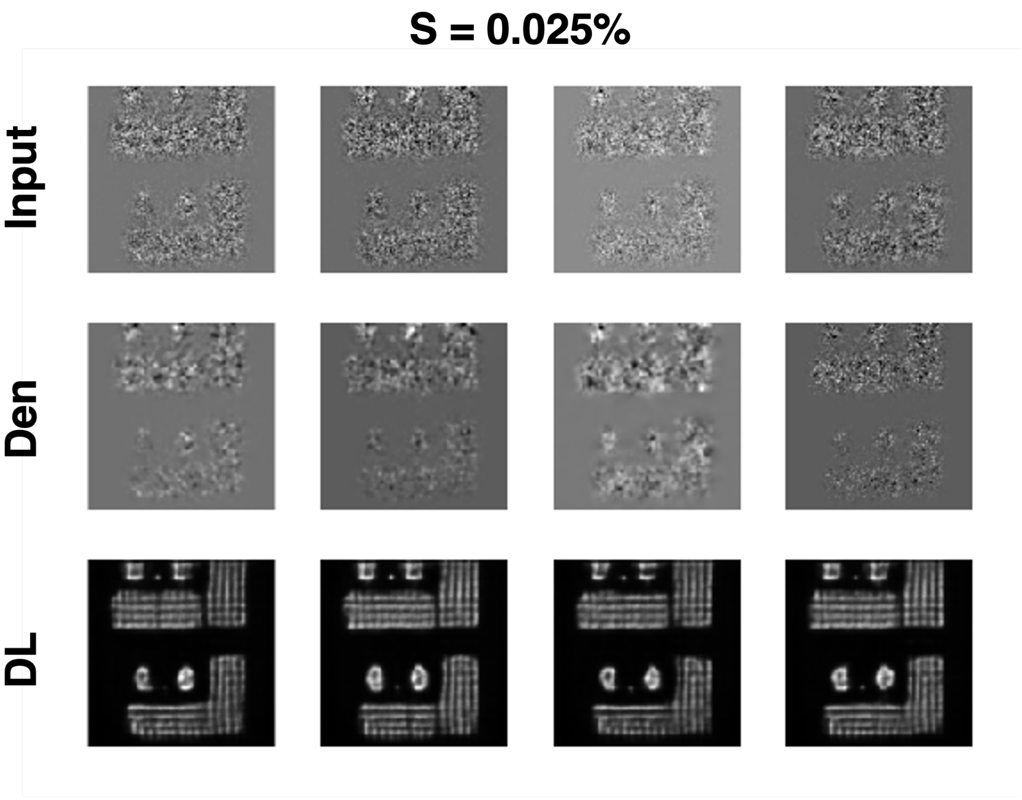

Figure S3: Experimental results for 4 different noisy images of the test sample taken with a sampling rate of S=0.025%.

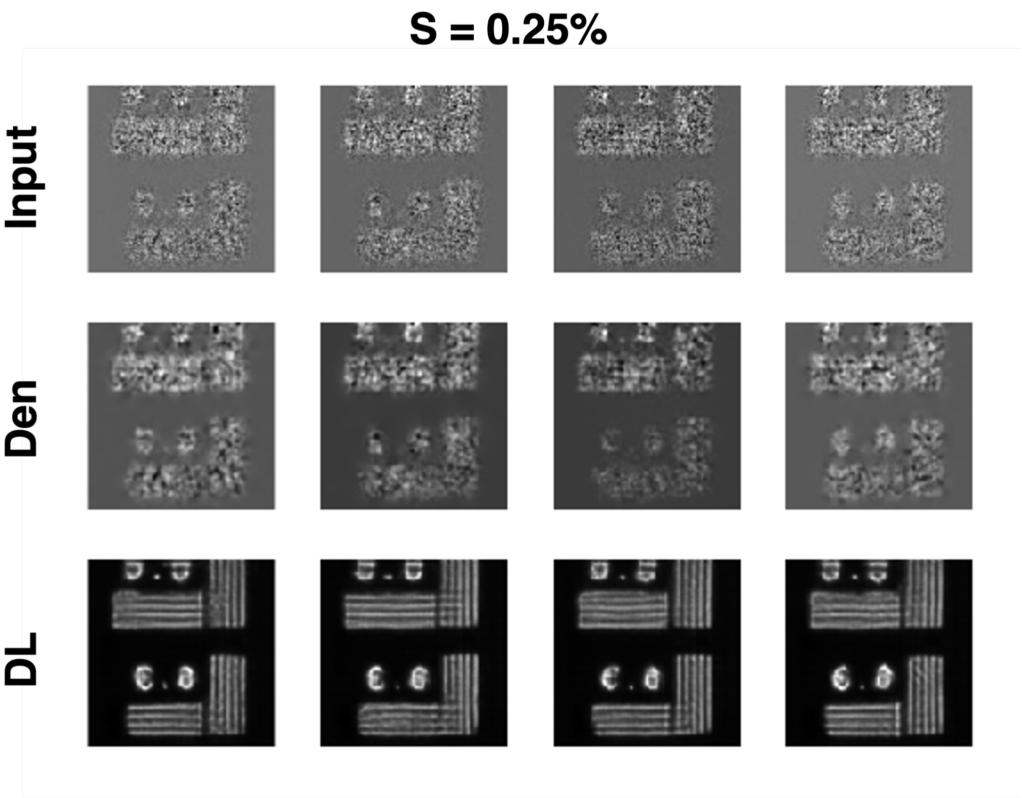

Figure S4: Experimental results for 4 different noisy images of the test sample taken with a sampling rate of S=0.25%.

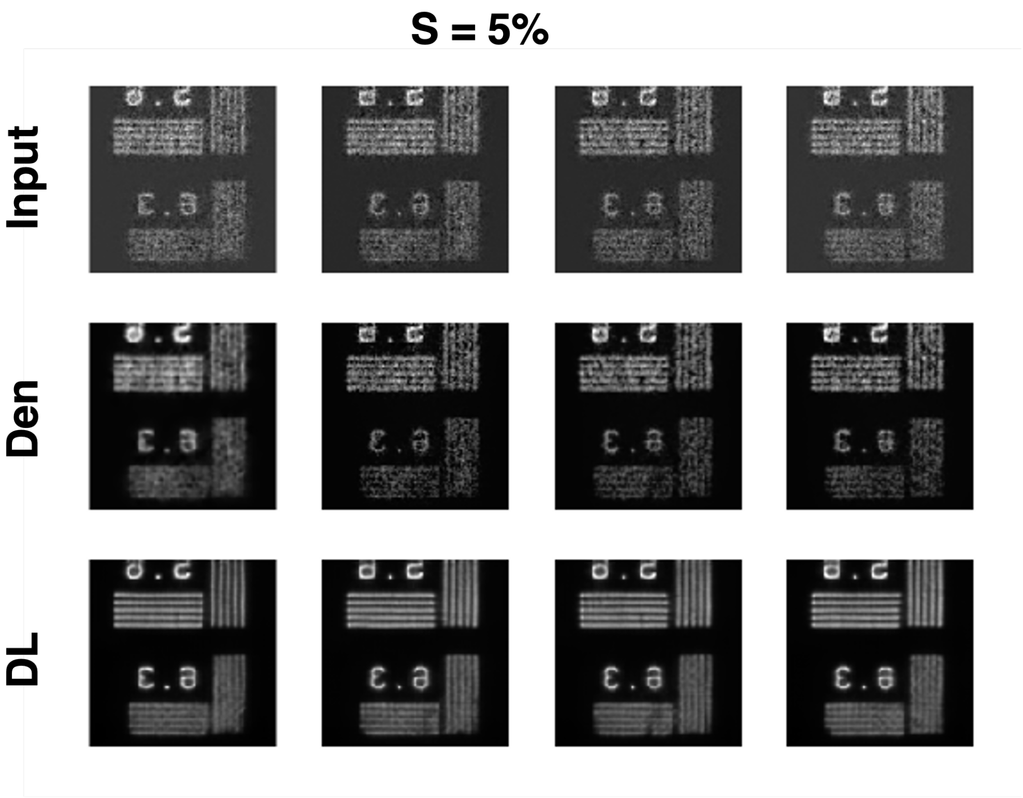

Figure S5: Experimental results for 4 different noisy images of the test sample taken with a sampling rate of S=5%.

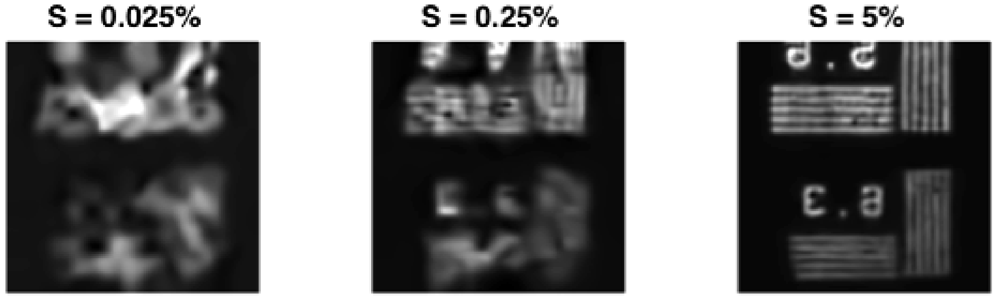

S	SSIM BL+G	SSIM BM3D	SSIM DL
0.025%	0.12 ± 0.02	0.17 ± 0.06	0.56 ± 0.01
0.25%	0.24 ± 0.04	0.57 ± 0.08	0.66 ± 0.01
5%	0.84 ± 0.02	0.90 ± 0.06	0.87 ± 0.02

Figure S6: The Block-matching and 3D filtering (BM3D) [1] algorithm has been taking into account to compare the results obtained with our Deep Learning Model. We used BM3D package developed for MATLAB and applied the filer to the same images used to test the DL model (*i.e.* 100 images for each sampling ratio) using a sigma value of the noise power spectrum (PSD) optimized on the expected result (our ground truth in Fig.2 of the main text). In this figure, three representative images of the BM3D output are shown, one for each sampling ratio. Although achieving significantly better results than the denoise used for our analysis in terms of SSIM, as shown in the attached table, the BM3D gives satisfactory results only in the case with a higher sampling ratio (5%), while fails for the cases with a lower sampling ratio S. Unlike the DL model, which utilizes artificial intelligence to reconstruct features not visible in the input image, the BM3D acts on the noise by actually cleaning the image only if the sampling ratio is such that correlated pixels are enough to retrieve a noisy image of the sample.

References

[1] Y. Mäkinen, L. Azzari, and A. Foi, "Collaborative filtering of correlated noise: Exact transform-domain variance for improved shrinkage and patch matching," *IEEE Transactions on Image Processing*, vol. 29, pp. 8339–8354, 2020.
